# Difficult-to-treat psoriatic arthritis: refining the definition using a statistical model in a real-life cohort

**DOI:** 10.3389/fmed.2024.1509082

**Published:** 2024-12-16

**Authors:** Ennio Giulio Favalli, Giacomo Biganzoli, Gilberto Cincinelli, Matteo Ferrito, Ester Luconi, Maria Manara, Martina Biggioggero, Patrizia Boracchi, Roberto Caporali

**Affiliations:** ^1^Department of Rheumatology and Medical Sciences, ASST Gaetano Pini-CTO Institute, Milan, Italy; ^2^Department of Clinical Sciences and Community Health, University of Milan, Milan, Italy; ^3^Department of Biomedical and Clinical Sciences, University of Milan, Milan, Italy; ^4^Data Science and Research Center (DSRC), University of Milan, Milan, Italy

**Keywords:** psoriatic arthritis, difficult-to-treat, b/tsDMARDs, predictive model, drug resistance

## Abstract

**Objectives:**

The study aims to evaluate the applicability of the D2T psoriatic arthritis (PsA) definition, adapted from rheumatoid arthritis, within a single-center observational cohort of PsA patients treated with b/tsDMARDs. In addition, we aimed to establish a numerical index defining D2T-PsA based on the ratio of observed to expected failed b/tsDMARDs and to develop a predictive model identifying features associated with the D2T condition.

**Methods:**

The study included 267 consecutive adult PsA patients receiving b/tsDMARDs, collecting demographic, clinical, and clinimetric data. The prevalence of D2T PsA patients was assessed using a proposed definition. We then developed a predictive model to assess treatment difficulty, utilizing PsA-normalized failed b/tsDMARDs. A generalized linear model was applied to identify clinical and demographic features associated with D2T PsA, employing a bagging procedure for robust variable selection, followed by univariate and multivariable analyses.

**Results:**

Among the 267 patients, only 8 of them (2.9%) met the proposed D2T PsA criteria. In a subset of 177 patients analyzed using the predictive model, 17.2% of them demonstrated higher treatment difficulty. Univariate analysis revealed associations between treatment difficulty and female sex, psoriasis pattern, fibromyalgia, and steroid therapy. Multivariate analysis confirmed significant associations between fibromyalgia, nail and pustular psoriasis, and steroid use.

**Conclusion:**

According to the predictive model, the proposed D2T PsA definition identified a small subset of patients with increased treatment difficulty. These findings highlight the need for refining the criteria to better define D2T PsA patients, providing valuable insights into managing complex treatment challenges in PsA.

## Highlights

- What is already known on this topic: although an approved D2T-PsA definition is still lacking, its translation from the D2T-RA definition captures only a fraction of the expected patient population due to the heterogeneous articular and extra-articular manifestations of PsA, which account for a unique D2T definition.
- What this study adds: we developed a predictive model that assessed the difficulty of treatment based on failed b/tsDMARDs normalized against expected failures to highlight disease-specific D2T features.
- How this study might affect research, practice, or policy: our findings suggest potential criteria refinement for defining D2T PsA patients, providing insights to better manage treatment complexities in PsA.

## Introduction

Psoriatic arthritis (PsA) is a chronic inflammatory disease with an extremely heterogeneous phenotype that may include musculoskeletal, skin, and systemic involvement and several comorbidities that often complicate the clinical picture. This diverse presentation contributes to the complexity of treating PsA, necessitating optimal management of all domains that constitute its clinical picture in each patient (1).

Treatment strategies for PsA have significantly improved with the application of treat-to-target and personalized medicine approaches, as well as the development of new drug classes, such as biological and targeted-synthetic disease-modifying anti-rheumatic drugs (b/tsDMARDs) (2–4). However, there is still a high proportion of patients who do not achieve adequate disease control or who experience secondary ineffectiveness or drug-related adverse events after an initial good clinical response (5, 6). Accordingly, real-life cohorts still include a significant proportion of PsA patients refractory to multiple targeted drugs (7, 8). In this scenario, managing these difficult-to-treat (D2T) patients still represents an unmet need and a significant challenge for the rheumatology community.

In 2021, the European Alliance of Associations for Rheumatology (EULAR) defined D2T rheumatoid arthritis (RA) (9), while international scientific societies have not yet elaborated on an official definition of D2T-PsA. A first attempt was made to adapt the criteria for D2T-RA to PsA, resulting in a preliminary definition from a single-center observational cohort that identified 33.9% of patients as D2T-PsA (10). However, being designed for a different disease, the definition of D2T-RA could only partially apply to a clinical scenario as complex and multifaceted as PsA. Building on the foundational work of Perrotta et al., various authors have defined the profile of D2T PsA patients, identifying varying demographic and clinical characteristics associated with this peculiar condition (11–14). Given the complexity of psoriatic disease, where a one-size-fits-all approach does not seem feasible (15), a recent review of the literature conducted to inform a research project on this topic revealed that a consensus on the definition of active disease and the number of failed therapies to define D2T PsA patients is still lacking (6).

Furthermore, the lack of any reference to the time frame in which the failures occurred within the natural history of the disease may undoubtedly represent a limitation in properly identifying D2T patients.

The study aimed to test the use of the preliminary D2T-PsA definition (10) within a single-center observational cohort of PsA patients treated with b/tsDMARDs. In addition, we aimed to identify a numerical index defining D2T-PsA based on the number of b/tsDMARDs failed compared to the number of b/tsDMARDS cycles, considering a (worse) scenario with a high rate of therapy failure.

## Materials and methods

### Patient selection

We conducted a retrospective study enrolling consecutive adult patients affected by PsA in a single-center, real-life setting between September 2022 and February 2023. The inclusion criteria were as follows: (a) age over 18 years old at the time of PsA onset; (b) a diagnosis of PsA according to CASPAR criteria or based on a rheumatologist’s clinical judgment; and (c) treatment with a b/tsDMARD for at least 3 months. All patients routinely visited our tertiary-level rheumatology outpatient clinic at Gaetano Pini-CTO academic hospital and treated with b/tsDMARDs according to the EULAR recommendations for managing PsA (3). Patients are primarily referred to our department for the articular and musculoskeletal manifestations of the disease. However, the choice of therapies is made according to the overall clinical picture, taking into account both cutaneous and other non-musculoskeletal manifestations, even in the case of well-controlled arthritis. All patients had been previously enrolled in a longitudinal observational registry (Ethics Committee approval: 138_1999).

Patients’ demographic data, including age, sex, smoking habit, and body mass index (BMI), were collected at the diagnosis. Age at PsA diagnosis, PsA phenotype (collected at the last visit as the pattern of the patient’s record of articular manifestations as peripheral, axial, peripheral+axial, or enthesitic), diagnosis of skin psoriasis and type of cutaneous involvement (plaque, scalp, nail, palmoplantar, or guttate psoriasis), and history of dactylitis, uveitis, or inflammatory bowel disease (IBD) were collected as clinical data. Moreover, the presence of comorbidities was noted, including symptomatic osteoarthritis, fibromyalgia, depression, cardiovascular comorbidities, hyperlipidemia, diabetes mellitus, and a history of malignancy.

At the last visit, laboratory data, including C-reactive protein (CRP) levels, were collected alongside clinical assessments such as tender and swollen joint counts (TJC-68 and SJC-66, respectively), patient and physician global assessments (PGA and PhGA) on a 0–10 scale, the Visual Analogue Scale for pain (VAS pain) on a 0–10 scale, the number of tender entheseal sites based on the Leeds Enthesitis Index 0–6 (LEI), the percentage of body surface area (BSA) affected by psoriasis, the Health Assessment Questionnaire-Disability Index (HAQ-DI), the Rheumatic Disease Comorbidity Index (RDCI) score, and the Disease Activity in PSoriatic Arthritis (DAPSA) score.

We collected information regarding the number and mechanisms of action of previous and current b/tsDMARDs, the current or prior use of csDMARDs, current corticosteroid therapy, and dosage. Due to the lack of data on the mechanisms of failure of previous bDMARDs from previous clinical records in a substantial proportion of patients or lines of therapy, reasons for bDMARDs failure were not recorded.

### Identification of D2T patients according to the previously proposed definition

**Figure 1:**
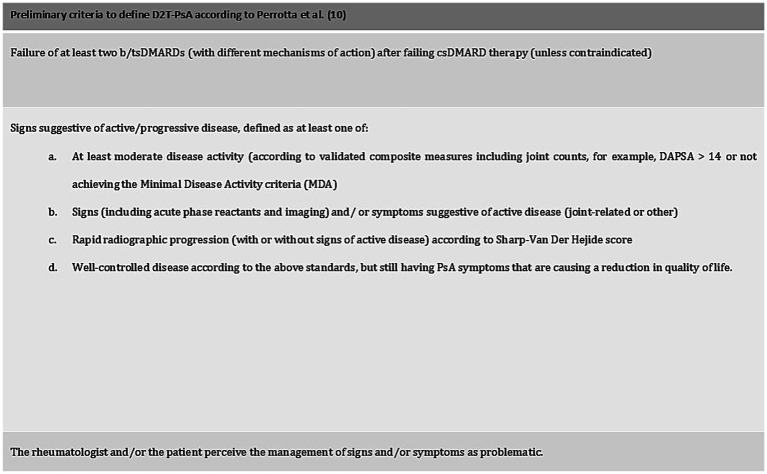
Preliminary criteria to define D2T-PsA. All three criteria should be fulfilled to define a D2T-PsA patient.

First, we evaluated how the preliminary definition performed in identifying D2T patients. Perrotta et al. proposed potential criteria to define D2T-PsA, taking a cue from the EULAR definition of D2T-RA (10), as reported in Figure 1.

The univariate association between the condition and the clinical characteristics of the patients was explored, exploiting logistic regression with penalized maximum likelihood to account for the small proportion of difficult-to-treat patients. The association was presented with OR and its 95% confidence intervals.

### Development of an index proxy of the treatment difficulty

**Figure 2:**
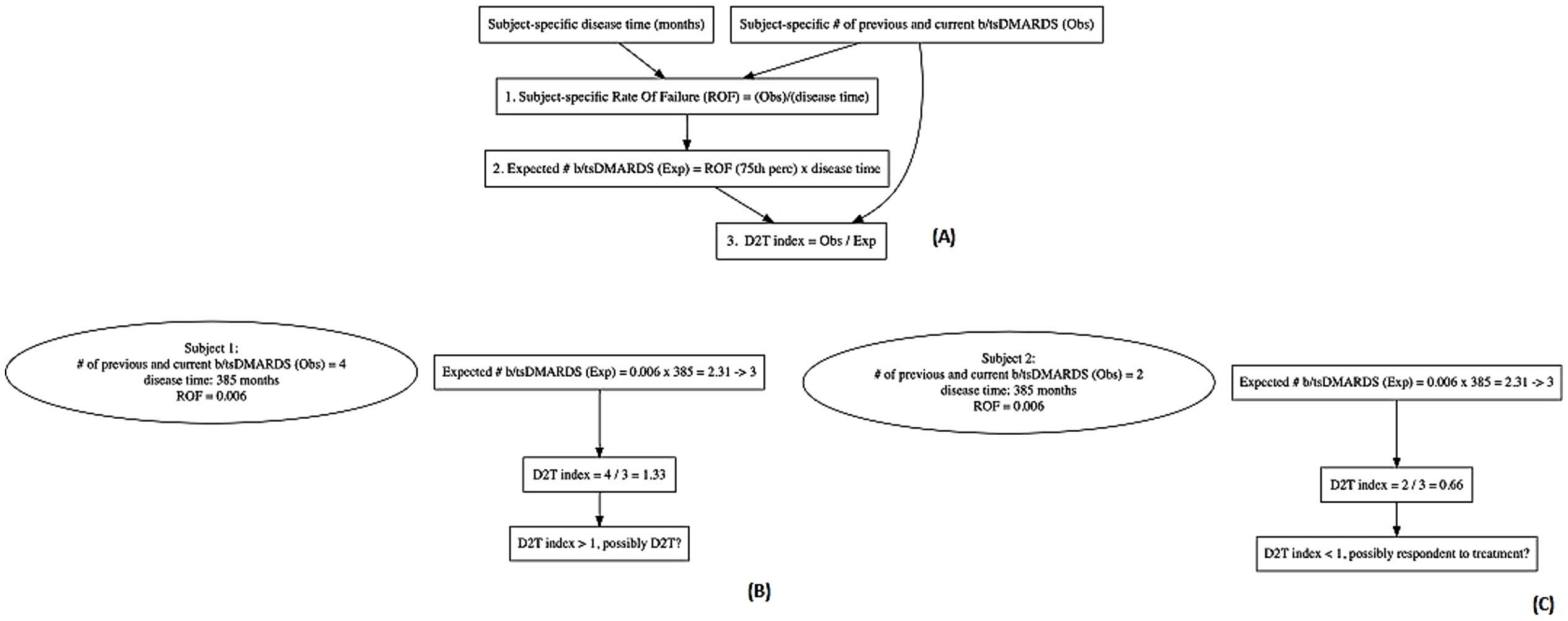
Schematic representation of the computation of the difficult-to-treat index with two clinical examples of a patient possibly difficult to treat and a patient possibly respondent to treatment. **(A)** Steps of development of the proxy of the D2T status. **(B)** Example of a subject with a high D2T status, and **(C)** example of a subject respondent to treatment according to the D2T index.

The primary indicator for the treatment difficulty was the count of previous b/tsDMARDs failed by the patient, computed by adding one unit from the number of previous b/tsDMARDs to consider the present therapy. The subject-specific actual count of b/tsDMARDs was divided by the length of the subject-specific disease time to obtain a rate of failure (ROF) of a therapy (*rate = actual number of b/tsDMARDs/months of follow-up time*) (Figure 2A—step 1). Then, the 75th percentile of the distribution of the failure therapy rate was taken as a cut-off value to identify patients requiring the highest number of b/tsDMARDs needed to manage the disease. The cut-off was then used to obtain the expected number of b/tsDMARDs for each patient as follows (Figure 2A—step 2):

$\begin{array}{l} \text{expected number of}\;b/\text{tsDMARDs}=\\ \text{rate}75th\;x\;\text{months of follow}-\text{up}\;\text{time} \end{array}$

The resulting expected number was rounded up to an integer. The ratio between the actual and expected number of b/tsDMARDs was considered a proxy of a patient’s treatment difficulty (Figure 2A—step 3). A ratio > 1 indicates an individual as potential D2T patient (e.g. subject 1 in Figure 2B), while a ratio < 1 indicates a subject respondent to treatment (e.g. subject 2 in Figure 2C). Moreover, the higher the value, the higher should be the complexity of the individual to be treated.

### Delineation of patient characteristics related to treatment difficulty–univariate analysis

To assess the relative difficulty in treating PsA, we employed a generalized linear model with a Poisson distribution of the model error. The model’s linear predictor was linked to the response variable with a *log* link function. The actual number of b/tsDMARDs was the response variable with an offset term added to account for the expected number of b/tsDMARDs, achieved by log-transforming the expected number of b/tsDMARDs. The variables explored for a potential association with treatment difficulty were the following: sex, PsA subset, presence of psoriasis, psoriasis type, obesity (BMI >30), cigarette smoking, history of dactylitis, concomitant osteoarthritis, fibromyalgia or IBD, corticosteroid use, DAPSA, PGA, and HAQ score. Categorical variables were included as dummy coding, and results are reported as the ratio between the treatment difficulty of each category and the reference one. For continuous variables, non-linear effects were accounted for by transforming the variables with a restricted cubic spline transformation and testing the statistical significance of the non-linear regressor terms in the model with Wald statistics.

For numerical covariates, an estimated effect >1 indicated an increase in the relative difficulty as the variable increased. For categorical covariates, an estimated effect >1 indicated a higher relative difficulty for a category than the reference one.

### Delineation of patient characteristics related to treatment difficulty–multivariable model

To retrieve the majority of robust variables among all the covariates in terms of association with the difficulty of a subject to be treated, a backward variable selection procedure based on the Akaike Information Criterion (AIC) was performed. To this aim, observations with incomplete data for all the covariates were necessarily omitted. A thousand independent samples with replacement from the data were obtained to ensure the robustness of the selection of the covariates from the backward variable selection procedure.

Variable selection was applied in each sample, and the relative frequency of selection of a specific variable in the model was computed.

Nonetheless, all variables were included in the final model with an unbiased and weighted estimate of the regression coefficient, reflecting the mean of the distribution of each regression coefficient calculated in the backward-selected model across all iterations. If the variable was not selected by the backward procedure, the coefficient value would be considered zero. The ratio between the actual and expected number of b/tsDMARDs was finally predicted by the final model. Individuals were distinguished into two groups depending on their predicted ratio (≤1 or > 1). Finally, a nomogram considering the linear predictor of the final model was produced to represent the contribution of the clinical and demographic characteristics that lead to higher (or lower) levels of the treatment difficulty. All the statistical analyses were performed with R software (4.2.2).

## Results

### Cohort characteristics

A total of 267 adult PsA patients were recruited, including 127 women (47.6%) and 140 men (52.4%), with a median age of 53 years (46–61) and a median disease duration of 154.5 months (IQR 89.0–249.25). Peripheral arthritis was the most common disease subset, observed in 71.2% of patients, while 205 patients (78.5%) had skin psoriasis. The majority of patients were affected by only one subset of psoriasis, predominantly plaque psoriasis (65.2%), while 39 (14.6%) patients presented two or more different subsets. Approximately one-fourth of patients were active smokers (17.5%) and 29 (10.9%) of patients were obese (BMI > 30). Fibromyalgia and osteoarthritis were diagnosed in 69 (25.8%) and 42 (15.7%) patients, respectively. The median (IQR) values for DAPSA, PGA, and HAQ were 4.2 (1.11–9.25), 2.00 (0.00–4.00), and 0.12 (0.00–0.38), respectively. The general characteristics of the cohort are listed in Table 1.

**Table 1:** Characteristics of the cohort.

*N*	267
Gender = M (%)	140 (52.4)
Age (median [Q1, Q3])	53.00 [46.00, 61.00]
PsA-predominant subset	
Axial (%)	29 (10.9)
Peripheral (%)	193 (72.3)
Axial Peripheral (%)	37 (13.9)
Enthesitis (%)	12 (4.5)
Presence of psoriasis (%)	205 (78.5)
Plaque psoriasis (%)	166 (81.4)
Scalp psoriasis (%)	36 (17.6)
Nail psoriasis (%)	30 (14.7)
Palmo plantar pustulosis (%)	17 (8.3)
Guttate psoriasis (%)	2 (1.0)
Obesity (BMI > 30) (%)	29 (11.1)
Smokers (%)	70 (27.5)
Presence of osteoarthritis (%)	69 (25.8)
Fibromyalgia (%)	42 (15.7)
History of dactylitis (%)	40 (15.0)
IBD (%)	4 (1.5)
Corticosteroid use (%)	31 (11.6)
Dosage (milligrams of prednisone equivalent) (median [Q1 – Q3])	0 [0–2.5]
DAPSA (median [Q1, Q3])	4.20 [1.11, 9.25]
PhGA (median [Q1, Q3])	0.00 [0.00, 1.00]
HAQ (median [Q1, Q3])	0.12 [0.00, 0.38]
RDCI (median [Q1, Q3])	0.00 [0.00, 1.00]
Number of previous b/tsDMARDs (median [Q1, Q3])	0.00 [0.00, 1.00]
Combination therapy with csDMARDs (%)	91 (34.1)
Months since PsA diagnosis (median [Q1, Q3])	154.50 [89.00, 249.25]
Actual *vs* expected number of b/tsDMARDs ratio (median [Q1,Q3])	1.00 [0.67, 1.25]
D2T patients, according to Perrotta et al.	8 (2.99%)

PsA: Psoriatic arthritis; BMI: body mass index; IBD: inflammatory bowel disease; DAPSA: disease activity in psoriatic arthritis; PGA: patient’s global assessment; HAQ: health assessment questionnaire; b/tsDMARDs: biologic/targeted synthetic disease-modifying anti-rheumatic drugs; RDCI: Rheumatic disease comorbidity index; D2T: difficult-to-treat.

### Proportion of D2T patients according to the previously proposed definition

Among 267 PsA patients enrolled, only eight patients (3%) met the D2T definition. Six D2T patients were women (75%), 6 (75%) had a peripheral subset of PsA, while 2 (25%) suffered from a predominantly axial disease. The mean age of D2T PsA patients was 57.3 years, with a mean disease duration of 203 months. Six patients (75%) suffered from skin psoriasis, whereas no D2T PsA patients had a BSA >3%. 50% of them suffered from fibromyalgia, and five patients (62.5%) complained of symptomatic osteoarthritis.

In univariate analysis, compared to non-D2T, D2T PsA patients presented a higher rate of osteoarthritis (OR = 4.76; 95% C.I. = [1.23, 21.03]), fibromyalgia (OR = 5.75; 95% C.I. = [1.42, 23.19]), and therapy with steroids (OR = 8.45; 95% C.I. = [2.07, 34.56]). Furthermore, D2T patients presented significantly higher patient global assessment (PGA 0–10) (OR = 1.68; 95% C.I. = [1.20, 2.34]).

Notwithstanding, among non-D2T patients, 24 were in moderate disease activity (9.3%).

Due to the imbalance between the groups’ numerosity, multivariate analysis was not feasible.

### Delineation of patient characteristics related to treatment difficulty – univariate analysis

After applying our new index, 46 patients (17.2%) showed a ratio between observed and expected b/tsDMARDs higher than one.

In univariable analysis, sex, psoriasis type, concomitant fibromyalgia, corticosteroid use, DAPSA, PhGA, and HAQ score seem to be factors associated with greater treatment difficulty.

**Figure 3:**
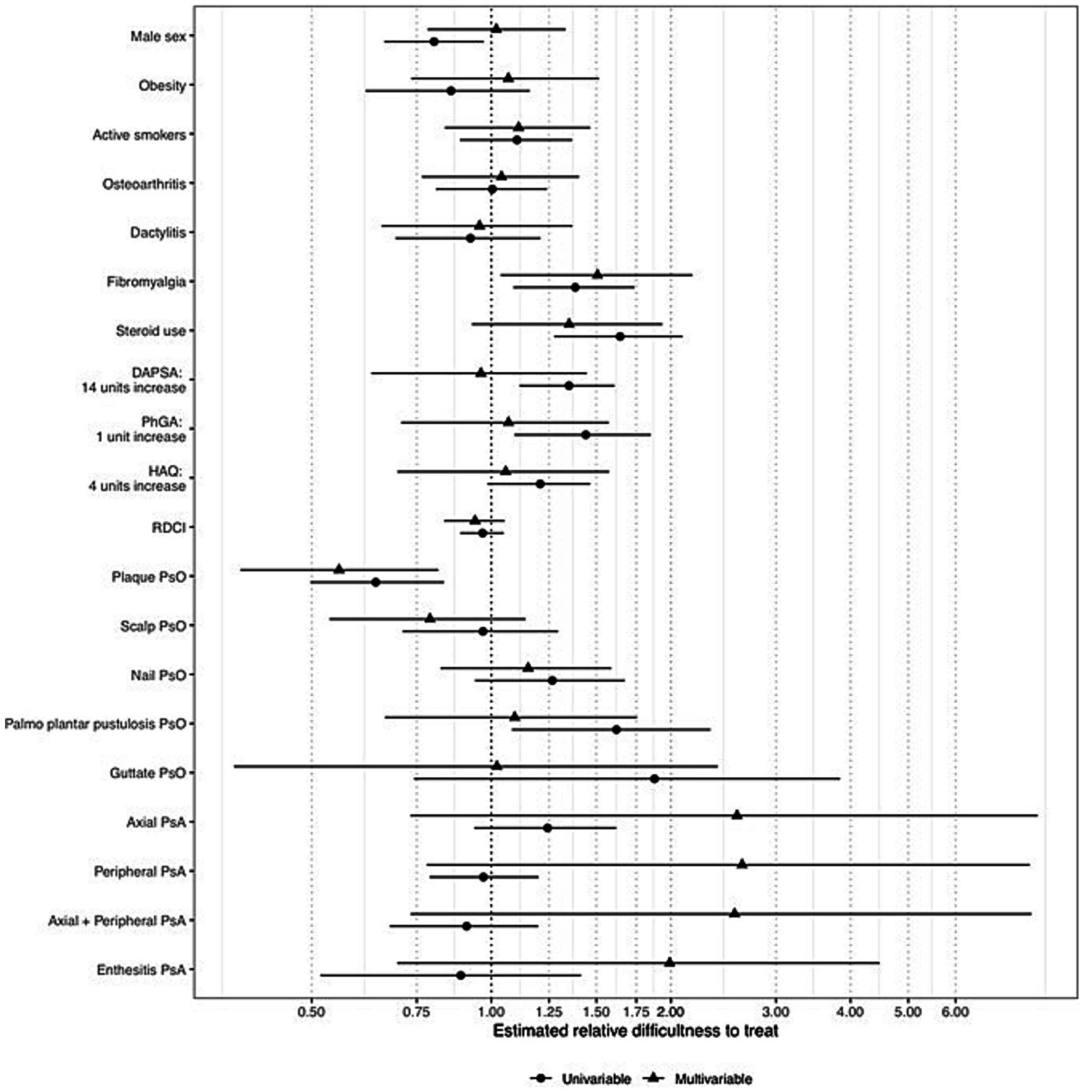
Unadjusted and adjusted estimates of the association of the covariates on the treatment difficulty. An estimate greater than one indicated an increase in treatment difficulty as the variable increased and vice versa. Notably, the closer to one of the estimates, the less indicative the covariate for treatment difficulty. DAPSA: disease activity in psoriatic arthritis; PhGA: physician’s global assessment; PsA: psoriatic arthritis; HAQ: health assessment questionnaire; PsO: psoriasis; PP: palmoplantar.

From the plot (Figure 3), increases in the PGA score, HAQ, or DAPSA appear to be significantly associated with an increased treatment difficulty. Also, fibromyalgia, corticosteroid use, psoriatic nail involvement, and palmoplantar psoriasis are all positively associated with an increased difficulty in treatment. On the other hand, the RDCI index and plaque psoriasis seem associated with lower levels of difficulty in treatment.

### Delineation of patient characteristics related to treatment difficulty – multivariable model

All the variables assessed in the univariate analysis were specified in the multivariable model as independent covariates. A total of 177 observations of over 267 had complete information for all the covariates and were used to fit the model. A trend of association with treatment difficulty was confirmed in the multivariable model for nail involvement, palmoplantar psoriasis, steroid use, concomitant fibromyalgia, HAQ, and PhGA, but not for DAPSA. Moreover, a more pronounced trend of association with higher levels of difficulty in treating osteoarthritis was evident. A trend of association with lower levels of treatment difficulty was maintained for plaque psoriasis and more evident for scalp psoriasis. Interestingly, steroid use, fibromyalgia, plaque psoriasis, and scalp psoriasis were also retained when the backward variable selection procedure based on AIC was applied.

The robustness of these results was confirmed with the resampling procedure. Through over a thousand perturbations (1,000 independent samples with replacement of the 177 observations), the factors chosen the most by the variable selection procedure based on AIC were plaque psoriasis, fibromyalgia, steroid use, and scalp psoriasis. Supplementary Table S1 represents the relative frequency of selection of each variable.

The histograms showing the distribution of the model regression coefficients derived from the 1,000 perturbations are shown in Supplementary Figure S1. A nomogram showing the contribution of each covariate in defining treatment difficulty is reported in Supplementary Figure S2.

**Figure 4:**
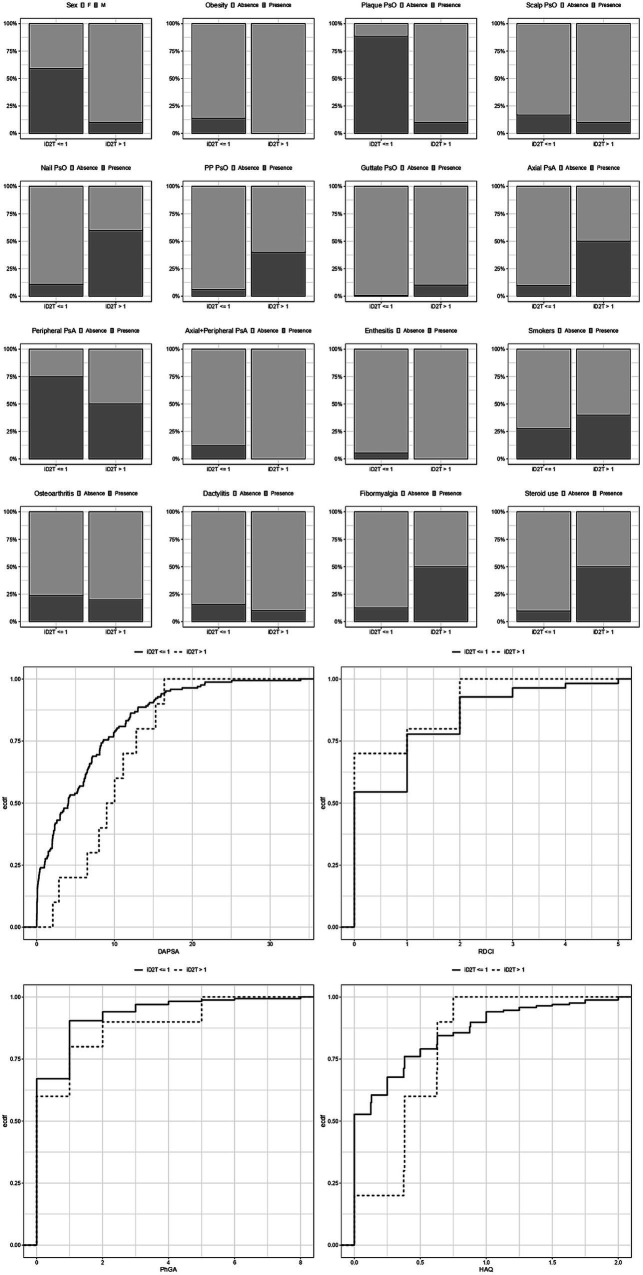
Distributions of each variable considered in the models conditioned to have a predicted ratio (iD2T) equal to or lower than one, greater than one (potentially difficult-to-treat individuals), or greater than 1.5 (highly potentially difficult-to-treat individuals). iD2T: difficult-to-treat index; PsO: psoriasis; PsA: psoriatic arthritis; IBD: inflammatory bowel disease; DAPSA: Disease activity in psoriatic arthritis; PhGA: physician’s global assessment; HAQ: health assessment questionnaire.

When the prediction of the actual-to-expected number of b/tsDMARDs ratio of each subject in the data with the final model was performed, 15 out of 177 individuals (8.4%) resulted in a predicted value >1. Figure 4 reports the distributions of each variable considered in the models conditioned to have a predicted ratio > 1 (potential D2T individuals) or equal to or lower than one.

As expected, potential D2T individuals include a greater proportion of female patients, smokers, patients with fibromyalgia, and steroid users. Interestingly, they are also characterized by higher DAPSA, HAQ, and PhGA scores when values above the median of the distribution are compared.

## Discussion

In our study, we aimed to assess the applicability of a proposed definition of D2T-PsA, which borrows its criteria from the EULAR D2T-RA definition. In our cohort, we observed an extremely low proportion of D2T-PsA patients, about 3% of our cohort, which is approximately 10-fold lower than that published by Perrotta et al. (10) in a similar analysis. This result was unexpected, as we anticipated a higher proportion of difficult-to-manage patients in our PsA cohort.

Hence, we aimed to study the effect of covariates on a numerical continuous D2T score, which differs from the mere presence of a condition by quantifying the association of each covariate with the degree of treatment difficulty.

Thus, we developed a statistical model to intercept prognostic factors associated with more complex management. Using this model, we identified 17.2% of patients as potential D2T-PsA. This proportion reduced to 8.4% when we applied the predicted actual-to-expected number of cycled b/tsDMARDs. The use of this model allowed us to investigate the role of demographic and clinical factors as possible markers of a D2T condition. Concomitant fibromyalgia, certain types of skin involvement, as well as corticosteroid use, were positively associated with a higher treatment difficulty. The result of our study only partially aligns with the proposed definition (10) and the results from Philippoteaux et al. (11). In detail, fibromyalgia was associated with the D2T status in both analyses, according to previous research depicting fibromyalgia as an unfavorable prognostic factor in terms of treatment response (8) and therapeutic target achievement (16–18).

On the other hand, in our study, obesity was not associated with D2T-PsA (10, 11, 14) despite evidence pointing at a high BMI as an obstacle, lowering the response to treatment with TNF inhibitors (TNFis) (19, 20) and the achievement of remission or minimal disease activity (21, 22). However, the link between obesity and poor treatment response to TNFis does not apply to other available mechanisms of action, with even an inverse association with IL-17 blockade, which showed a better clinical response in overweight and obese PsA patients (23).

In our study, we observed a robust positive association between more complex disease management and certain types of psoriasis, namely palmoplantar psoriasis and nail involvement, coherently with published data, showcasing how skin psoriasis, and in particular palmoplantar psoriasis, detrimentally affects patients’ quality of life (24). Similarly, cutaneous involvement in terms of extension of the skin disease was found to be associated with a difficult-to-treat condition in psoriatic arthritis patients in the work by Vassilakis et al. (14), although with no mention of the phenotype of skin psoriasis. Furthermore, our study confirms that psoriatic nail involvement imposes a higher burden of joint inflammatory disease (25) and that PsA patients who manifest both skin and joint disease have worse clinical outcomes than those with only musculoskeletal-related symptoms (8, 26).

These data underscore the deep groove between PsA and RA as clinical entities, ultimately advocating for a future PsA-D2T definition that embraces the multifaceted nature of PsA, overcoming the comfort of the D2T-RA shadow (15, 27).

Another distinguishing feature of our model compared to the previously proposed D2T definition is the criterion concerning the number of mechanisms of action a patient must fail to meet. Not all treatments for PsA are equally effective across different disease domains, meaning patients with multi-domain involvement may face a reduced therapeutic arsenal, potentially falling short of the D2T multi-refractoriness threshold (27).

In line with this, our exploratory study aimed to depart from the framework of the D2T-RA definition, shifting the critical element of our proposed model. Instead of centering a possible D2T definition solely on refractoriness to multiple b/tsDMARDs, we redefined refractoriness from a simple cumulative count of failed drugs to an algebraic construct. In this model, a higher ratio between observed and expected cycles through different therapies reflects greater management complexity, regardless of the number of mechanisms of action previously attempted.

This approach accounts for a future where PsA patients may receive therapies with multiple mechanisms of action. The crux of our definition lies in the relative urgency with which clinicians must switch or swap therapies. Consequently, even patients treated over extended periods with multiple drugs sharing a single mechanism of action (such as the previously limited options for TNFα inhibitors) can still be considered difficult to treat. From our perspective, this shift represents a necessary and pivotal evolution, redefining the core of the D2T criteria in a subtle yet meaningful way.

From a methodological point of view, it is important to note that we used the 75th percentile of the therapy-failure rate distribution to calculate the number of expected b/tsDMARDS. Adjusting this threshold would affect the sensitivity and specificity of the index. However, while the index is threshold dependent, the regression coefficients—expressed in terms of relative treatment difficulty—remain robust and reliably indicate varying degrees of treatment complexity.

Another noteworthy aspect of our index and definition is that a patient could qualify as D2T even in the absence of current signs and symptoms of active disease. This highlights the model’s focus on the overall therapeutic journey and management challenges, rather than solely on active disease manifestations.

Accordingly, a patient with a history of treatment failures in greater numbers than expected should be considered as D2T, even if, at the time of assessment, the disease is apparently in good control since the efficacy of the current therapy does not erase the non-response to previous therapies and the difficulty in identifying a treatment capable of producing an effective response. Moreover, a patient could experience sufficient control over his/her musculoskeletal manifestations while simultaneously suffering from poorly controlled psoriasis, inflammatory bowel disease, or uveitis, extra-articular manifestations often under-reported in many uni-dimensional indexes (28). Similarly, the achievement of a minimal disease activity state could still potentially leave room for up to two unhampered disease domains. Such a discrepancy between the actual disease domain activity and the composite indices score underscores a profound distance between RA and PsA.

Indeed, the centrality of the rapidity of therapy switching rather than the number of previously failed mechanisms is remarked upon also by the fact that in our cohort, the majority of difficult-to-manage patients do not reach the threshold number of failed mechanisms of action set by the definition proposed (10). At the same time, many of them meet the new definition we propose with our index. Even though our analysis did not focus on the reason for therapy failures, the need for a more rapid sequential switching of b/tsDMARDs could most likely be due to a succession of primary non-responses attributable to a biological mismatch between the drug target and the disease target (29), which theoretically identifies a more difficult-to-manage pattern than the same number of therapeutic switches observed over a longer time frame. Ultimately, in line with the path marked out in the D2T-RA field (30), Lubrano et al. provided different definitions for patients failing therapies due to persistent residual inflammation (“refractory-to-treatment PsA”) and for those with suboptimal disease control due to the overall management of the inflammatory and non-inflammatory components of the disease (“D2T-PsA”) (31). Although the importance of exploring the presence of residual inflammation in complex diseases is of paramount importance in the treatment of complex cases, our model does not offer discrimination between difficult-to-treat patients with or without persistent inflammatory disease, thus refraining from any inappropriate distinction between difficulty-to-treat and refractoriness-to-treat in our cohort.

Although our study expands the understanding of the D2T-PsA concept, several limitations must be acknowledged. First, the relatively low sample size from a monocentric cohort and the study’s cross-sectional design partially limit the generalizability of the results. In addition, potential patient selection bias should be considered, as the majority of the PsA patients at our center are “articular-predominant,” with less severe extra-articular manifestations. Therefore, independent longitudinal studies with broader populations are warranted to validate our findings and better identify treatment modifiers, encompassing the multifaceted nature of psoriatic syndrome.

Moreover, while we employed a statistical model to identify patients with higher treatment difficulty, this approach is currently feasible only in research settings. In standard clinical practice, such a model would likely be too cumbersome to implement. Given the exploratory nature of our study, more studies are needed to confirm the prognostic value of the factors identified in this study as potentially associated with treatment difficulty. Future studies could refine the statistical model into a practical scoring algorithm for D2T PsA, making it more accessible for routine clinical use.

Nevertheless, it is important to emphasize the exploratory nature of our study, which aims to explore the prognostic factors best suited for identifying truly difficult-to-manage PsA patients. To achieve this, we adapted and reinterpreted the D2T-RA criteria, focusing on factors uniquely related to the psoriatic disease spectrum. Although our study does not provide sufficient evidence to establish a definitive D2T-PsA definition, it offers valuable insights suggesting that the D2T-PsA criteria should diverge from the D2T-RA framework, as we believe is necessary.

## Data Availability

The datasets presented in this article are not readily available because the datasets used and/or analyzed during the current study are available from the corresponding author upon reasonable request. Requests to access the datasets should be directed to Gilberto Cincinelli, gilberto.cincinelli@unimi.it.
