# Supplementary material for: Difficult-to-treat psoriatic arthritis: refining the definition using a statistical model in a real-life cohort
Source: Front Med (Lausanne). 2024 Dec 16;11:1509082. doi: 10.3389/fmed.2024.1509082 (PMC11682903; doi:10.3389/fmed.2024.1509082)
Supplement: Supplementary file 1 [file Data_Sheet_1.docx]

**Supplementary Table S1. Relative frequency in which a variable is selected in the AIC backward selection procedure.**

| **Variables** | **Relative frequency of selection** |
| --- | --- |
| Plaque psoriasis | 0.887 |
| Fibromyalgia | 0.784 |
| Steroid use | 0.528 |
| Scalp psoriasis | 0.525 |
| Peripheral PsA | 0.497 |
| Axial PsA | 0.474 |
| Axial Peripheral PsA | 0.446 |
| Enthesitis | 0.384 |
| Smoker | 0.316 |
| Nail psoriasis | 0.302 |
| RDCI | 0.302 |
| PhGA | 0.302 |
| Palmo plantar pustulosis PsA | 0.264 |
| Osteoarthritis | 0.134 |
| Dactylitis | 0.134 |
| DAPSA | 0.123 |
| Sex | 0.113 |
| HAQ | 0.098 |
| Guttate psoriasis | 0.088 |
| Obesity | 0.03 |

**Abbreviations:** PsA: Psoriatic arthritis; RDCI: Rheumatic Disease Comorbidity Index; PhGA: Physician Global Assessment; DAPSA: Disease Activity in Psoriatic Arthritis; HAQ: Health Assessment Questionnaire.

**Supplementary Figure S1. Distribution of the model regression coefficients derived from the 1000 perturbations.** Some distributions are centered to zero, indicating that the factor does not have a robust association with the response. Some others have a clear distribution centered on positive or negative values, indicating robust positive or negative associations to the response.

**
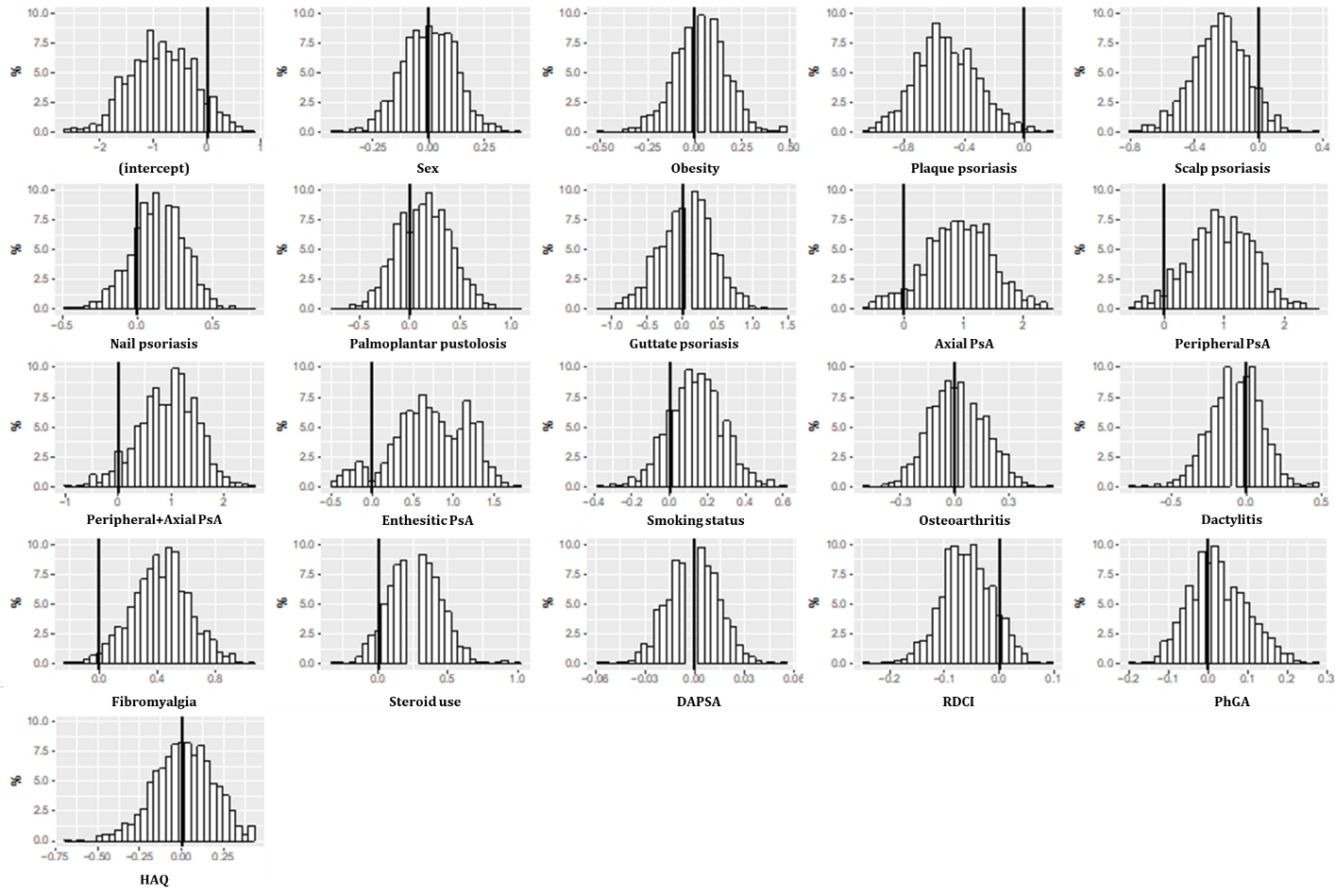
**

**Abbreviations:** PsA: Psoriatic arthritis; DAPSA: Disease Activity in Psoriatic Arthritis; RDCI: Rheumatic Disease Comorbidity Index; PhGA: Physician Global Assessment; HAQ: Health Assessment Questionnaire

**Supplementary Figure S2. Nomogram reporting the contribution of each variable in the definition of**

**the difficultness-to-treat of a subject.**

Consider the Points value associated with the value of the single clinical or demographic characteristic of a hypothetical PsA subject. Sum each value to have a Total Points score. Find the value of the linear predictor and of the Difficultness-to-treat corresponding to the Total Points value.


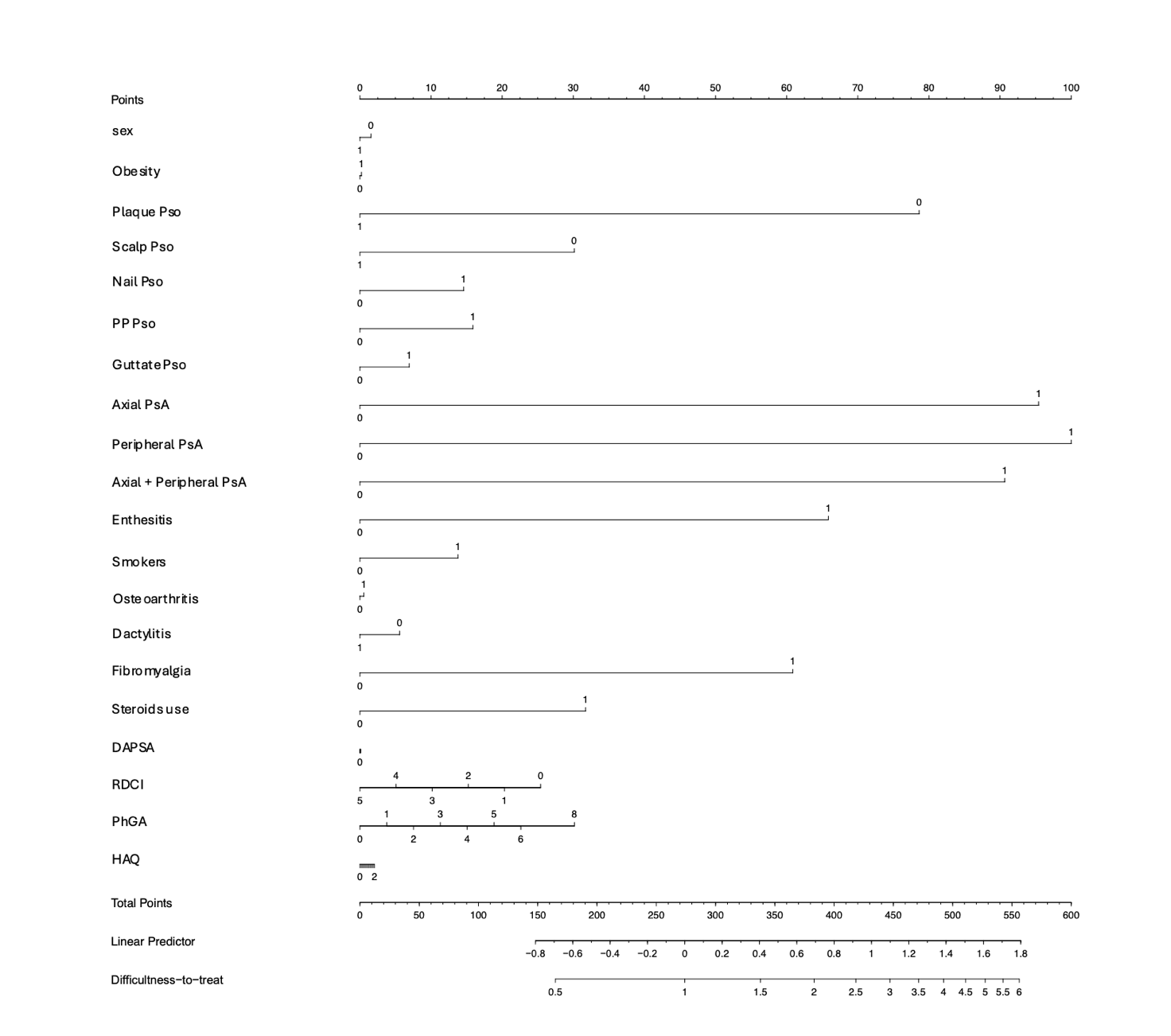


As an example, we will consider the characteristics of a hypothetical subject to show the calculation of the score of the Difficultness-to-treat according to the nomogram.

Based on the value of each variable, we will find the correspondence points in the nomogram. The correspondence point is obtained with the help of an imaginary vertical line that starts from the value of the variable for the subject in the correspondent line and terminates in the points line:

Sex=Male (1), so 0 points.

Obesity=Yes (1), so 0 points.

Plaque psoriasis=Yes (1), so 0 points.

Scalp psoriasis=No (0), so about 30 points.

Nail psoriasis=No (0), so 0 points.

Palmo plantar pustulosis=No (0), so 0 points.

Guttate psoriasis=No (0), so 0 points.

Axial psoriasis=No (0), so 0 points.

Peripheral psoriasis=Yes (1), so 97.5 points.

Axial+peripheral psoriasis=No (0), so 0 points.

Enthesitis=No (0), so 0 points.

Smoker=Yes (1), so about 15 points.

Osteoarthritis=No (0), so 0 points.

Dactylitis=No (0), so about 6.25 points.

Fibromyalgia=No (0), so 0 points

Steroids use=1 (Yes), so about 32.5.

DAPSA=0, so 0 points.

RCDI=1, so about 21.2 points.

PhGA: 0, so 0 points

HAQ=0, so 0 points.

Now we can calculate the total score with the sum of the points: 30+97.5+15+6.25+32.5+21.2

=202.45. This value in the total points line corresponds to linear predictor of about -0.47 (this value is found with an imaginary vertical line that starts from the 202.45 in the total point line). The patient is not difficult to treat.
